# Voles and weasels in the boreal Fennoscandian small mammal community: what happens if the least weasel disappears due to climate change?

**DOI:** 10.1111/1749-4877.12388

**Published:** 2019-07-29

**Authors:** Hannu YLÖNEN, Marko HAAPAKOSKI, Thorbjörn SIEVERT, Janne SUNDELL

**Affiliations:** ^1^ Department of Biological and Environmental Science and Konnevesi Research Station University of Jyväskylä Jyväskylä Finland; ^2^ Lammi Biological Station University of Helsinki Lammi Finland

**Keywords:** cascading effects, climate change, least weasel, population cycles, predator–prey

## Abstract

Climate change, habitat loss and fragmentation are major threats for populations and a challenge for individual behavior, interactions and survival. Predator–prey interactions are modified by climate processes. In the northern latitudes, strong seasonality is changing and the main predicted feature is shortening and instability of winter. Vole populations in the boreal Fennoscandia exhibit multiannual cycles. High amplitude peak numbers of voles and dramatic population lows alternate in 3–5‐year cycles shortening from North to South. One key factor, or driver, promoting the population crash and causing extreme extended lows, is suggested to be predation by the least weasel. We review the arms race between prey voles and weasels through the multiannual density fluctuation, affected by climate change, and especially the changes in the duration and stability of snow cover. For ground‐dwelling small mammals, snow provides thermoregulation and shelter for nest sites, and helps them hide from predators. Predicted increases in the instability of winter forms a major challenge for species with coat color change between brown summer camouflage and white winter coat. One of these is the least weasel, *Mustela nivalis nivalis*. Increased vulnerability of wrong‐colored weasels to predation affects vole populations and may have dramatic effects on vole dynamics. It may have cascading effects on other small rodent–predator interactions and even on plant–animal interactions and forest dynamics.

## INTRODUCTION

Anthropogenic climate change has created many stressors that threaten wild populations failing to adapt to novel conditions. In places where there is typically snow in winter, the effects of climate change on species may be driven by altered snow regimes (Penczykowski *et al.* 2017). Snow cover affects the life of ground-dwelling animals and food webs in many ways. Importantly, snow creates thermoregulative shelter and an insulated subnivean space, further providing a physical and visual refuge from predators. The duration and end of snow cover also drive phenology through natural selection in many animal species (reviewed by Penczykowski *et al.* 2017).

Approximately one-third of the world's land area is covered by snow during winter (Lemke *et al.* 2007). The need for understanding how altered snow regimes impact food webs is particularly urgent because climate change is occurring most rapidly in regions of the world that historically have had cold, snowy winters. A good example of how altered snow regimes is driving phenology is camouflage mismatch in seasonally colored molting species confronting unpredictable snow cover. In northern Europe, there are 6 species of birds and mammals changing from summer pelage to a white winter coat: the mountain hare (*Lepus timidus* Linnaeus, 1758), the arctic fox [*Vulpes lagopus* (Linnaeus, 1758)], 2 arctic grouse species [*Lagopus lagopus* (Linnaeus, 1758) and *Lagopus muta* (Montin, 1781)] and 2 small mustelids (*Mustela nivalis nivalis* Linnaeus, 1766 and *Mustela erminea* Linnaeus, 1758). The combination of coat color change and climate change cause a dilemma; the late and unpredictable onset of snow cover and its earlier melting threatens the survival of animals with the wrong coat color compared to the background. For example, the weekly survival of snowshoe hares with mismatched fur to the background decreases up to 7% due to predation (Zimova *et al.* 2016).

Small mammals, either as prey for carnivores or as consumers of plants or insects, are a key component of the boreal ecosystem. In Finland, small mammals consist of grass-eating *Microtus* species and more forest-dwelling *Myodes* species (Sundell & Ylönen 2008) and insectivorous shrews (*Sorex* spp.). Microtine rodents are known to fluctuate in 3–5-year cycles (Hanski *et al.* 2001). Besides affecting food webs as consumers, many other species are affected by changes in rodent density. For instance, ground-nesting forest grouse populations decline when vole numbers crash after peak years because predators focusing on voles change to alternative prey (Angelstam *et al.* 1984).

The least weasel is suggested to be the strongest single factor shaping the multiannual dynamics of boreal voles (Korpimäki *et al.* 1991; Hanski *et al.* 2001). The predator–prey interaction between weasels and voles is of great importance in vole life history in different phases of the population cycle. During the population increase after the crash, the predation pressure is not intense as the numbers of specialist predators increase with a delay of half a year (Sundell *et al.* 2013). During the peaks of vole abundances, predation pressure is high, and during the decline or crash of the populations, extremely high, as the ratio between weasels and voles increases strongly in favor of predators. The dramatic impact of weasels alone on the mortality of voles during the population crash was documented by Norrdahl and Korpimäki (1995) in a radio-telemetry field study where the mortality rate of radio-collared voles caused by weasels was close to 80% of all study voles.

From the vole survival point of view, the intense predation pressure by weasels means increased alert and the need for early recognition of weasel presence in the home area. This is possible for the voles using the body odors and scent markings excreted by anal glands of weasels, which, like in all mustelids, are strong (Brinck *et al.* 1983). It is recognizable as a cue for increased predation risk in the hunting terrain of weasels by prey animals (Ylönen & Ronkainen 1994; Ylönen 2001; Apfelbach *et al.* 2005). Weasel–vole contact normally occurs in shady or dark cavities, under grass and shrubs, in holes under stones or tree-trunks, as well as under the snow in winter. Here the visual sense is not important but the olfactory is: both for weasels to smell prey (Ylönen *et al.* 2003) and for voles to recognize risk through the presence of weasels early enough and to respond accordingly, by fleeing or hiding (Sundell & Ylönen 2004).

Weasel have a high energy demand and in order to breed, female weasels require high enough rodent densities close to the nest to be able to feed the litter or to survive during the cold season (Haapakoski *et al.* 2013). Due to its small size, weasels can hunt voles and shrews under the snow in winter. However, their small body size makes weasels themselves vulnerable to attacks by a range of different avian and larger mammalian predators (Korpimäki & Norrdahl 1989). As the vole populations decline, the resident avian predators may also target their hunting on small predators like weasels. This may be especially true during autumn, as food for most animals becomes scarce. In addition, when vegetation withers, hunting weasels may be more visible to avian predators. Furthermore, the onset of winter and camouflage mismatch may expose weasels to avian predation (Atmeh *et al.* 2018).

Climate change is causing unpredictability of the onset and end of winters and has the potential to threaten the viability of specialist predator species, possibly leading to cascading trophic effects at the ecosystem level (Terraube *et al.* 2015). Specialist predators can have a major impact on food webs. A good example of this kind of species is the least weasel (hereafter weasel), which is a specialist predator of small mammals. The reason why this weasel is so important is that it is thought to regulate small mammal populations such as those of voles and shrews in Scandinavia (Hanski *et al.* 2001).

In this review paper we aim to illustrate the predator–prey interaction between voles and their major predator, the least weasel, in the northern boreal environment of Fennoscandia. We focus on both vole ability to recognize the increased risk and to behaviorally respond, in the evolutionary arms race, to weasel risk in breeding or survival adaptations. We then include the predicted climate change scenario to the vole–weasel interaction. This may alter the population dynamics of both prey and predators, or more explicitly change the patterns in the regular multiannual cyclicity of vole populations. If the high impact of weasels in the multiannual pattern of vole dynamics (Hanski *et al.* 2001) were to change due to climate change, the decline or disappearance of specialist predators might dampen small mammal population cycles (Korpela *et al.* 2014). Dampened prey cycles theoretically could then drive small mammal specialist predator populations towards extinction (Millon *et al.* 2014), and the dramatic changes would have substantial cascading effects on the small rodent community and on their predator guild in the north.

## BOREAL VOLE CYCLE AND PREDATOR–PREY INTERACTION

Factors causing regular multiannual density fluctuations in organisms, so-called population cycles, have been the subject of intensive investigation and debate for almost 100 years (e.g. Stenseth 1999). One of the most studied species group has been small rodents living in the Northern Hemisphere. Especially regular and pronounced are cycles of voles and lemmings living in strongly seasonal environments. Many hypotheses have been proposed to explain these cycles. Presently, most popular hypotheses are related to biological extrinsic factors, such as food and predation (Hanski *et al.* 2001; Turchin & Batzli 2001), alone but also most recently combined with occurrence of pathogens and diseases (Huitu *et al.* 2003; Forbes *et al.* 2015).

Predation was long seen only as a factor prolonging the low phase of the cycle or deepening the crash. However, later, the different relative roles of various kinds of predators were recognized (Andersson & Erlinge 1977). In general, generalist predators' effect on prey cycles is thought to be stabilizing due to their habit of switching between prey types according to their availability. Similarly, avian predators, even though they might have specialized diets concentrating on small rodents, can stabilize population fluctuations of the prey because they can respond fast numerically by moving without significant delay from low prey populations to the sites with high prey availability over the vast landscapes. In this way, nomadic avian predators can also cause spatial synchrony of small rodent populations. In contrast, the resident specialist predators of small rodents, which are often small carnivorous mammals, especially the small mustelids the weasel and the stoat, cannot travel long distances and are highly dependent on local prey availability. They tend to have a destabilizing effect on prey populations (Hanski *et al.* 1993). These animals cannot respond as fast numerically as avian predators, and their response is likely to involve a time lag.

Avian predators and generalist predators are more common and numerous in southern areas than resident predators, meaning that resident predators' role is relatively larger in the north. This is commonly linked to the increasing gradient observed in cycle length and amplitude from south to north (Hanski *et al.* 1991). This gradient is also associated with climate with longer duration of snow cover and snow thickness in the north, which gives partial protection from many non-specialized predators (Hansson & Henttonen 1985).

### The special role of the smallest carnivore in the world: the least weasel

Common resident vole specialists in the north are small mustelids, the stoat and the weasel. The northern subspecies of weasels, the least weasel, which is also the smallest carnivore mammal in the world, is thought to be the most important predator of voles and lemmings. Weasels cause overwhelming mortality of populations, especially in declining vole populations (Norrdahl & Korpimäki 1995). This species has characteristics that make it a real threat to voles.

The least weasel is unique among carnivores because of its fast reproductive potential. Female weasels can have 2 litters per breeding season, in favorable conditions even 3, and occasionally they may breed in winter outside of their normal breeding season. Young females can mature in the same breeding season they have been born and produce a litter of their own. Litter sizes can be large, on average 6–10, but litters of 14 have been observed (Sundell 2003). Even if the least weasel has high reproductive capacity, they cannot cope with their prey, voles, which are even more efficient in reproduction. Because of this, weasel numbers often following those of their prey with a time lag, in theory a necessary condition for classic predator–prey cycles to occur (May 1973).

The least weasel is about the size of its main prey, meaning that it has a very restricted diet containing almost no other prey items than small rodents (Korpimäki *et al.* 1991). Their small size and slender body shape mean they have a high surface to volume ratio, resulting in high heat loss and high energy needs. Thus, the least weasel needs a relatively large amount of food even for maintaining its basic metabolic level (Gillingham 1984). The small-sized weasel can enter the tunnels and cavities of voles, whether they are under ground or snow, making it a very efficient predator in all seasons compared to other vole predators. Weasels can also enter the nests of voles and eat the pups. It cannot store much energy as fat as it needs to be slim to follow voles into their refuges, and, therefore, it kills more than is required for its immediate needs whenever possible, and stores the excess food for later use (Oksanen *et al.* 1985; Jędrzejewska & Jędrzejewski 1989). However, the least weasel prefers fresh food and, therefore, its kill rate can be much higher than its consumption rate and its effect on vole populations is higher than can be concluded just based on energy needs and weasel numbers. Because of its specialized diet, the least weasel is observed to have type II functional response, leading in theory to unstable dynamics and even cycles in its prey population (Sundell *et al.* 2000).

Its small size and the fact that the least weasel lives in the same habitat as its prey exposes it to other larger vole predators. In fact, the least weasel is often observed to be preyed upon by avian predators. This happens more often when vole numbers are declining and weasels need to move more to find the remaining prey (Korpimäki & Norrdahl 1989). In the snowy season this means that weasels have to move more on top of the otherwise protective snow cover.

## VOLE PERSPECTIVE IN THE ARMS RACE BETWEEN VOLES AND WEASELS

### Recognition of fear

In the tunnels and cavities on the ground and in the darkness under the snow, weasels hunt using olfactory sense. In an experiment using the Y-maze, Ylönen *et al.* (2003) clearly showed that weasels preferred to enter the maze branch providing odor cues of either bank voles [*Myodes glareolus* (Schreber, 1780)] or field voles [*Microtus agrestis* (Linnaeus, 1761)] over a branch with clean vole cage bedding at the end of the tube. Similarly, voles or other small rodent prey try to receive correct information on the presence and vicinity of a predator using the smell left by moving predators. Most mammalian predators have a typical smell, which they use for their own social communication, which is commonly excreted from anal glands via urine and/or feces, or through body rubbing (Erlinge *et al.* 1982). These predator social scents, widespread throughout all mammalian taxa, have common sulfur and/or nitrogen compounds (Apps *et al.* 2015), which are perceived to our nose as strong and sticky. Prey species, like voles in our case, can use these odor cues as a measure of risk by mammalian predators, and decrease their activity, including diel activity, foraging and even reproductive activities (Ylönen & Ronkainen 1994; Ylönen *et al.* 2006; Sundell *et al.* 2008; Haapakoski *et al.* 2013, 2015).

It is almost trivial to state that "Olfaction is a central aspect of mammalian communication, providing information about individual attributes such as identity, sex, group membership or genetic quality" (Weiß *et al.* 2018, p. 420). We know a lot about the importance of olfactory sense in mate choice from mice to men: on the role of, for instance, major histocompatibility complex in mate quality recognition and avoidance of inbred matings (Wedekind *et al.* 2000). Also in food selection, olfaction is of essential importance (Nevo *et al.* 2015). However, in most experiments, using the odor of predators as the mean of manipulation of the risk of predation, we tend to use very rough and broad mixtures of predator scents. This is especially true in larger-scale field studies where bedding of small mustelid cages is commonly distributed in the environment of study voles (e.g. Mappes & Ylönen 1997; Fuelling & Halle 2004; Trebatická *et al.* 2012). In contrast, studies dealing with single synthetic odor components out of the big bouquet of natural odors have problems finding the components carrying a biological meaning (Apfelbach *et al.* 2015; Sievert & Laska 2016).

Just recently we have started to think that animals receiving important, life-saving odor information of predation risk from the environment must be as accurate as the odors used in social communication and mate choice. Biological odors are organic materials, which have normal metabolism and dilution paths in time. Thus, predator scent that is left behind should be recognized by prey animals and provide accurate information about not only who was there but also when (Bytheway *et al.* 2013). Small mustelids like stoats and weasels do not have large ranges (Erlinge & Sandell 1986; Gehring & Swihart 2004). If there is enough rodent prey, they are typically resident to a certain area and visit in certain time intervals prey patches they had been visiting previously (Erlinge & Sandell 1986). During these visits, the risk for voles is high, but in between the visits low. Thus, aging of predator scent, disappearance of odor compounds in time, and the effects of different aged odors on prey response need to be studied more as there are only a few relevant studies so far (Hegab *et al.* 2014; Sánchez-González *et al.* 2018).

## Predator scent or the smell of an acute fear shock by an actual predator?

A growing field of exploration and experimental testing in mammalian predator–prey interactions is olfactory intra-species communication via so-called alarm pheromones. Alarm pheromones or "Schreckstoff" (von Frisch 1938) are used for communication across phyla (Bowers *et al.* 1972; Boissy *et al.* 1998; Beale *et al.* 2006). It is generally assumed that alarm pheromones serve as a warning signal within a colony/group or family for social species of insects, fish and mammals (Breed *et al.* 2004; Kiyokawa *et al.* 2004; Gomes *et al.* 2013). Several publications have succeeded in describing the chemical properties of alarm pheromones for different groups of invertebrate species (Bowers *et al.* 1972; Howe & Sheikh 1975; Kuwahara *et al.* 1989). However, there is so far little work on mammalian alarm pheromones. Brechbühl *et al.* (2013) discovered in strains of laboratory rats (Wistar) and mice (C57BL/6J and OMP-GFP) that their alarm pheromones are structurally similar to predator odors.

Contrary to predator-based odor cues, vertebrate prey species do not habituate to alarm pheromones (Hutchison & Marvin 1995; Hartman & Abrahams 2000). This could be caused by different information transferred in those components. While predator odors could inform about a general predator presence in the area, alarm pheromones are only released after a successful escape, signaling an immediate risk in the area.

There is ongoing debate about whether the chemicals currently referred to as alarm pheromones are true pheromones in a strict sense (Magurran *et al.* 1996; Viney & Franks 2004), but there is no argument about the elicited behavioral response. As the methods for chemical analysis have massively improved in the past decade, it will only be a matter of time before the true nature of these "odors of stress" is revealed.

## Do voles respond to olfactory weasel risk cues and to live weasels similarly?

There are ample studies, as well as experiments and information, demonstrating that small rodents use the predator scent as a measure of risk of predation. How accurately we do not know yet, but, in general, the responses are plausible and enhance prey vole probabilities of surviving over a risky period. Reviews by Lima and Dill (1990), Ylönen (2001) and Apfelbach *et al.* (2005, 2015) depict how prey animals perceive risk of predation and respond to experimentally increased risk. The normal responses are either freezing or fleeing, staying still or seeking shelter where predators would not be able to enter. There seems to be a dichotomy in either doing nothing or doing something very rapidly. Both seem to be better anti-predatory adaptations than moving a bit or slowly, where the prey individual only attracts predator attention and possibly provokes an attack.

In our own experiments we were able to verify the dichotomy in bank vole movements and choice of selecting a hole for escape. Some voles stayed at the site where they recognized the presence of a weasel and froze. If running to a hole, the voles did not take the risk of getting stuck in too small a hole but selected the next larger one, which was easier to enter (Sundell & Ylönen 2004). By this means the escaping voles may have escaped from larger male weasels at least, if not the smaller female voles. In a study where the living environment of voles and also weasels was experimentally fragmented consisting of the same amount of protective tall grass habitat, but either in one large or 4 small patches, the vole trappability decreased, especially in the continuous habitat where the weasel was living inside the same large patch as the voles were. The presence or visit of a weasel forced the voles out of the protective habitat to a risky matrix area without protective vegetation (Haapakoski *et al.* 2012, 2013).

Two most common vole species in Fennoscandia and much of Europe, the field vole and the bank vole, inhabit different habitats; field voles as herbivores are grassland specialists and granivorous bank voles inhabit the forests (Sundell & Ylönen 2008). Bank voles have a 3-D habitat and they use trees for foraging buds and lichens. Do bank voles use trees to escape when chased by a predator, and, if yes, how effective is climbing as an escape strategy? We tested the escape reactions of grassland specialists, field voles, and forest species, bank voles, when escaping weasels (Mäkeläinen *et al.* 2014). Both species did not mind using the offered possibility to climb to a tree when there was no weasel present. When chased by a weasel, 14 out of 51 tested bank voles climbed the tree and only 2 out of 30 field voles climbed. Weasels followed the horizontal tube of escape; that is, most field voles would have been falling prey to weasels as 25% of the bank voles escaped successfully. Forest-dwelling, climbing bank voles survive population decline better than ground-dwelling, clumsier and non-climbing field voles, which are suggested to be preferred prey by weasels in the multi-species prey vole guild.

The activity of voles is dependent on predator activity. If predators, like owls, are nocturnal only, the prey animals adjust their activity to dusk and dawn to avoid the peak activity of owls (Jacob & Brown 2000). In the nocturnal predator–prey interaction, a full moon decreases prey activity as it exposes them more to avian predation, which is documented in desert rodents, with no grass cover allowing shelter during moonlight (Brown *et al.* 2001; Kotler *et al.* 2010). Boreal bank voles have polyphasic activity patterns throughout the day and night (Ylönen 1988, however see Bleicher *et al.* 2019). In a field study where we monitored boreal voles and weasels with radio-tracking in large enclosures, the voles carefully followed the resting times of weasels in their activity and decreased their activity as the weasels in the enclosure started to move again (Sundell *et al.* 2008). Seeking food and handling it after having found a profitable foraging patch are essential for animal energy gain, wellbeing and survival. In a simple and, for future research, influential optimality model, Brown (1988) suggested animal foraging efficiency and, thus, energy gain to be determined by foraging costs, predation costs and costs from other activities missed during foraging. He developed a method called giving-up-density (GUD), which provides the harvest rate of a food patch under different risk (cost) of predation. He proposed that the animals quit foraging as the profitability of a patch decreases and time to find food in the patch rises, increasing the time exposed to predation (predation costs). GUD measurements have become a standard in animal foraging ecology and decision-making under risk of predation (Bedoya-Perez *et al.* 2013). Decreased foraging under increased predation risk has been documented in dozens of studies with different taxa from small mammals to ibex, ungulates and porcupines (Brown & Alkon 1990; Kotler *et al.* 1994; Altendorf *et al.* 2001; Ylönen & Brown 2007). Optimality in foraging reflects fitness-related behaviors and survival strategies, and, thus, provides a far broader picture of animals' optimal behavior than only gaining food and energy to survive (Stephens *et al.* 2007).

Producing offspring under high risk of predation, especially targeted against the pups, is a strongly debated issue. The least weasel as a predator provides an excellent example in studying the effects of increased weasel risk on breeding of prey voles. The weasel is so small that it can enter almost any hole or nest of voles or other ground-dwelling or subterranean small mammals. Thus, it is an effective nest predator as well. As the vole populations decline, the numbers of adult and sub-adult prey voles decrease and weasels may be forced to seek the nests of last breeding females to find food. They might even be forced to enter the nests of ground-nesting or even hole-nesting passerine birds to exploit eggs of fledglings as food (Järvinen 1985).

What should a female vole in reproductive condition do under risk, where the probability of pups being killed by a predator, the weasel, is high? Furthermore, if the cues of the nest with pups lead weasels to the nest site, or if reproductive and lactating weasels attract olfactory hunting weasels, the female may lose her own life as well (Korpimäki *et al.* 1994; Ylönen & Ronkainen 1994). In a series of laboratory and semi-natural field experiments, we demonstrated breeding suppressive effects of weasel presence or weasel odors on reproduction of the bank vole and the field vole (Ylönen & Ronkainen 1994; Koskela & Ylönen 1995; Mappes & Ylönen 1997). However, more extensive field studies often did not verify any effects in breeding of voles during the best summer conditions, despite increased or simulated risk of weasel predation (Trebaticka *et al.* 2012, but see Fuelling & Halle 2004). In an overwintering study under weasel odor-simulated risk of predation, however, a significant effect and a delay of first reproduction of 1 month under weasel risk compared to start of breeding in populations with supplemental food and no weasel predation was again observed (Haapakoski *et al.* 2012). The results raise 2 essential questions: how the odor-based weasel cue persists in the breeding environment and if the recognition of risk needs to be happening at the onset the of breeding season. For the first question we have the answer that an odor remains a reliable cue under the snow, with no wind or rain that may dilute or fade the odor signal in summer. For the second question, we consider that if breeding has already started, stopping the breeding cycle seems to be difficult if not impossible. Running around in breeding condition but not breeding would possibly not bring any survival benefit anymore.

The partly contradictory studies may provide a synthesis in the form of variability of breeding strategies in animals: whether to invest in own survival and decrease breeding activity, suppressing or delaying reproduction (Ylönen 1994; Ylönen & Ronkainen 1994), along with other activities like moving and foraging. Alternatively, the second option is to invest in intensive reproduction, even with the risk of being the last one, with the hope that at least one pup will survive over the period of high risk (Duffield *et al.* 2017; Haapakoski *et al*. 2018; Sievert *et al*. 2019). The latter theoretical strategy, bet-hedging or terminal investment, has been documented in numerous taxa depending on either intrinsic factors like individuals' age or extrinsic threat factors for survival, like predation or parasitism (see tables 1 and 2 in the review by Duffield *et al.* 2017).

## WEASEL PERSPECTIVE: WEASEL AS SPECIALIST PREDATOR AND PREY

### Weasel behavior and survival adaptations along the vole cycle

Weasels have large reproductive potential if the food situation is good. This is true during the increase and peak years of vole cycles. Rapid reproduction with large litters is sustained by increasing population densities of voles. Weasels are regarded to be the strongest single factor in causing the vole numbers to turn from growth to decline. As the vole numbers are decreasing, the numerous weasels alone are sufficient to complete the crash leading to very low numbers of voles. Especially during autumns of high-density years preceding the decline, weasels are known to kill more prey than required for their daily energy needs. This phenomenon, called surplus killing, is, according to current knowledge, an adaptive behavior: hoarding food for future in the cold seasons (Jędrzejewska & Jędrzejewski 1989) when the caches do not rot rapidly. The same occurs in the smallest owl species, the pygmy owl (*Claucidium passerinum* Linnaeus, 1758), which hoards birds and small rodents in holes or nest boxes just before the onset of winter (Solheim 1984; Mappes *et al.* 1993).

During the milder season, the least weasel needs on average a bit more than one 25-g vole per day. The energy needs of female and male voles are equal despite their body size differences; during pregnancy, the female needs 3 times the amount of food that a non-breeding female needs (Macdonald 1995). During autumn and towards the winter, the energy needs and hunting efforts of weasels double (Haapakoski *et al.* 2013). The mortality effect of weasels from the population peak to decline and population low is dramatic. It is illustrated by the fact that during the peak phase of both prey voles and weasels, the number of weasels is estimated to be a maximum of 5–10 individuals per $km^2$. At the same time, the number of field or sibling voles can reach up to 10 000 voles per $km^2$ (Macdonald 1995).

After the vole population crashes, weasels are in trouble. As small vole specialists, they are not able to find alternative prey in the same manner as larger carnivores. If the vole populations start to decline during autumn and remain low until spring, the weasels need to put more effort and energy into hunting the decreasing and rare voles left. This also means more hunting trips, and more movement out of the sheltering ground vegetation or on the snow. Here come the top predators, raptors, owls and larger mammalian predators, as actors into the evolutionary play where the weasel partly changes from predator to prey.

### Fatal coat color dilemma along climate change

Predicted climate change scenarios suggest fundamental changes, especially in the winters of northern boreal areas. The environment is changing, but day length remains the same. The weasel changes its white winter coat in autumn based on a physiological mechanism of melatonin synthesis. The most important factor driving molting in mammals is a hormonal cascade induced by photoperiod (Zimova *et al.* 2018), which stays the same regardless of onset of snow cover. Plasticity of winter fur molt in weasels is very limited (Atmeh *et al.* 2018). For thousands of years, shortening of day length and onset of winter have been in strict correlation but not necessarily anymore. This makes weasels vulnerable to climate change-caused mismatch in color molting so that they are no longer camouflaging with the background color due unpredictable snow cover. Recently it has been found that climate change is affecting weasel mortality in Poland due to camouflage mismatch (Atmeh *et al.* 2018). In the Polish study area, both weasel subspecies were present, the northern least weasel, which changes coat color in winter, and the southerly common weasel (*Mustela nivalis vulgaris*), which remains brown during the winter. The relative proportion of white-coated least weasels is decreasing in the 2-weasel community (Atmeh *et al.* 2018). This means that climate change will strongly influence the mortality of the weasel due to prolonged camouflage mismatch at both ends of winter.

How will climate change impact weasels? Both onset and end of winters are predicted to be more often snow-free and the number of snow-coved days is decreasing (Atmeh *et al.* 2018). The key question is the extent of flexibility or polymorphism in the response of weasels to day length trigger, Zeitgeber, for starting the melatonin synthesis leading to coat change (Mills *et al.* 2018). Through polymorphism, natural selection can operate in favor of individuals which change their winter coat later, or which use cues other than day length as a trigger for the change. Mills *et al.* (2018) found in a global survey of 8 coat changing species polymorphic zones which could represent the material for evolutionary rescue for these species under climate change. However, the Fennoscandian least weasel, the key species impacting cyclic dynamics of several vole species, seems to have a very limited plasticity in the timing of coat color change (Atmeh *et al.* 2018).

Thus, least weasel survival, population numbers and dynamics on population dynamics of voles is an important issue. Through Central Sweden and the Baltic states there is an overlapping zone of 2 weasel subspecies, the more northern and eastern least weasel and the Central European common weasel. The former changes coat color and the latter does not. Thus, one scenario is the spread of the common weasel more to the north if environmental change favors brown remaining predators and least weasel plasticity or degree of polymorphism is limited (Atmeh *et al.* 2018). Spread of new species towards the north along the milder climate conditions is occurring. However, the main groups of immigrants, birds and insects have wings and can rapidly respond to environmental change. Mammals are slower and hindered by physical barriers like the Baltic Sea in invading Fennoscandia.

## CONCLUSIONS: WEASEL FAITH, VOLE COMMUNITIES AND FOREST LANDSCAPES

There exists a general picture that something weird is going on in vole cycles around Europe (e.g. Hörnfeldt *et al.* 2005; Millon *et al.* 2014) and that these changes reflect habitat changes, predator fluctuations and food webs in general (Penszykowski *et al.* 2017). The general assumption is the impact of climate change, for instance change in the North Atlantic Oscillation and its effects on winter properties, is a strong factor behind the general dampening of cycles. Korpela *et al.* (2014), examined the role of specialist and generalist predators, especially that of weasels in summer and winter dynamics of northern boreal voles in Finland. Their assumption was that there would be a strong climate driven effect on vole populations, especially during the winter. However, the extensive analyses showed a reverse picture: that weasel impact was strong during summer, and that winter conditions were not driving population collapses during the following summer.

The Europe-wide dampening of population cycles in mainly grassland species, *Microtus* voles and *Microtus*-like *Myodes* voles, the grey-sided vole (*Myodes rufocanus* Sundevall, 1846) (Hörnfeldt *et al.* 2005; Cornulier *et al.* 2013). Evidence on the interaction between climate predation and forest dwelling species like the bank vole is scarce. There are 2 Fennoscandian examples of drastic changes in vole dynamics, the temporal disappearance and return of vole cycles in Finnish Lapland (Henttonen *et al.* 1987; Cornulier *et al.* 2013) and the low densities of grey-sided voles in Sweden (Hörnfeldt *et al.* 2005) (Fig. 1.). Both seem to have as a common factor: the changes in land use and forestry, and, therefore, in landscape structures. However, the processes leading to vole cycle dampening and the magnitude seem to be different.

In Lapland, the change in the forest age structure was followed by disappearance of synchrony between the species in population peaks and crashes and especially the drastic decline of field voles. Along with the field vole decline, numbers of weasels declined and the stoat (*Mustela erminea*) became the major “regulator” of vole dynamics, however concentrating on field voles and field vole-type tundra voles and grey-sided voles. This allowed the competitor bank vole populations to grow and bank vole numbers remained high and stable over long periods (Henttonen 2000). As the field vole returned to the system, weasels and cycles returned as well.

Dampening of the Swedish grey-sided vole cycle is more clearly attributed to climate change (Hörnfeldt *et al.* 2004) and change in forest structures, like changes in the stony structures of forest floor and forest fragmentation (Ecke *et al.* 2006; Magnusson *et al.* 2013), without a direct link to small mustelid predation or *Microtus–Mustela* interaction.

Here we would like to add the direct link of winter change and its possible effects on small mustelid numbers to the abovementioned factors affecting the population dynamics of voles. Both weasels and stoats change their camouflage, providing protection from other predators during the snowy season. The white coat changes more or less at the same time in autumn, regardless of the timing of the onset of the snowy season. In addition, the brown coat appears back in spring, regardless of when the snow melts. If the degree of polymorphic flexibility in the coat change is small, both weasels and stoats are exposed to predation at both ends of win-

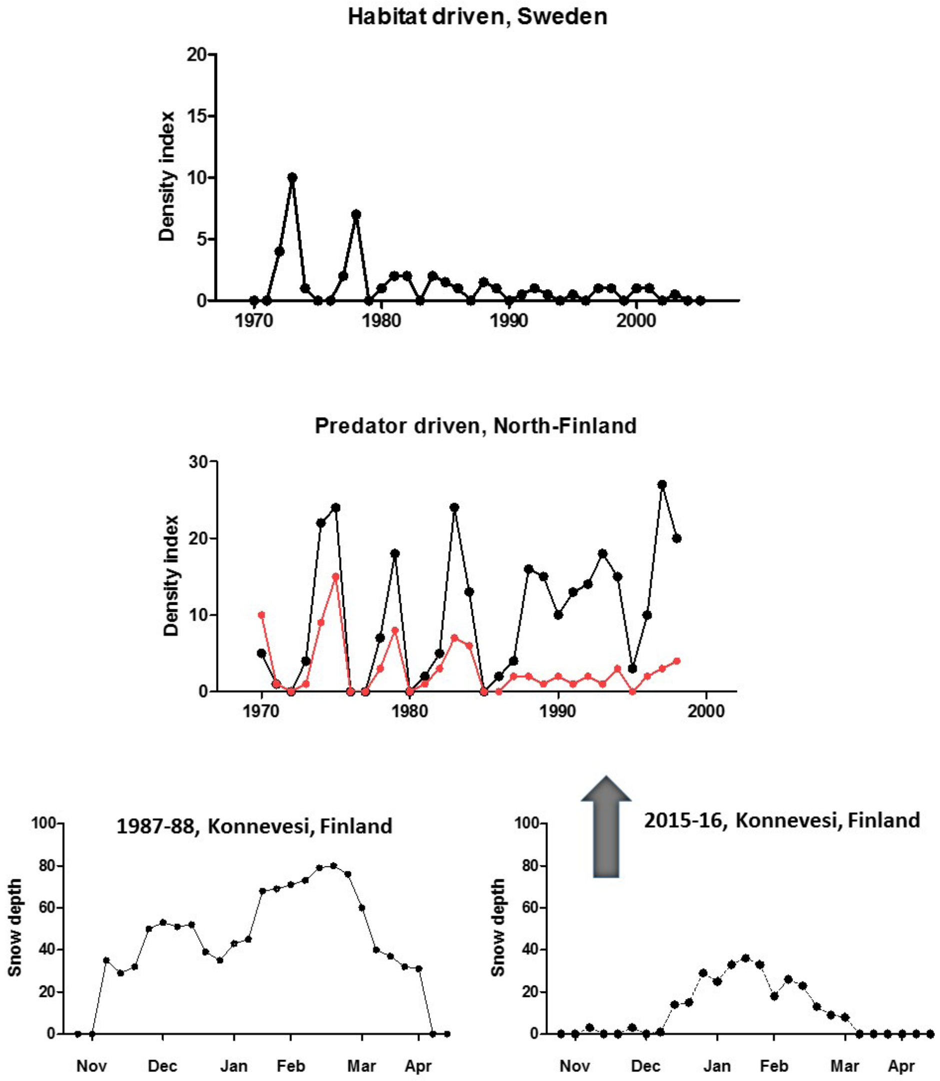

**Figure 1** Schematic presentation of 2 types of dampening of the Fennoscandian cyclic vole fluctuations. The grey-sided vole declines and stable low numbers are suggested to be due to habitat changes, especially in old pine forests (Hörnfeldt *et al.* 2005). The disappearance of synchronous cycles in Lapland (Henttonen 2000) is more clearly predator-driven. The disappearance of field voles (red line) had a cascading effect in releasing growth of competing bank vole populations, that stabilized at a high level not seen before (black line). The lower panels describe examples of winter change at Konnevesi, Central Finland: on the left the "old stable and snow-rich" winter and on the right the "current unstable wet winter," with late onset and early melting of snow cover. If this type of winter affects weasel survival as suggested, this could lead to similar dynamics disturbance in vole populations as observed in Lapland after weasel disappearance (indicated with the arrow).

ter. These are times that all animals, resident avian and mammalian predators included, in a strongly seasonal environment need more energy, for winter survival and for reproduction and feeding the spring-born young.

If small mustelids, and particularly the least weasel, decline or even disappear, this can cause drastic changes in vole dynamics, as indicated by the Lapland case (Henttonen 2000) but in a far larger geographical scale. If the type of dynamics change is driven by release of predation pressure by vole specialists, the populations may be stabilized to a permanently higher level than with a strong small mustelid predation. As alone the age structure change and fragmentation in forest structures cause disturbance in vole population dynamics, the effect of increasing seedling pest numbers may change forestry in a way not seen before. The disappearance of the world's smallest carnivore, the least weasel, from the northern boreal small mammal community could have dramatic effects on the mammal community and the landscape.

**Cite this article as:**

## Cite this article as:

Ylönen H, Haapakoski M, Sievert T, Sundell J (2019). Voles and weasels in the boreal Fennoscandian small mammal community: what happens if the least weasel disappears due to climate change? *Integrative Zoology* **14**, 327–40.
